# The Management and Diseases of the Dog

**Published:** 1881-10

**Authors:** 


The Management and Diseases of the Dog. By John Woodroffe Hill, F.R.C.V S. With Thirty-nine Illustrations. New York: William R. Jenkins. 1881. Pp. 383.

The author, in his preface, speaks very truly regarding the little attention that has been paid to the diseases of the dog. Man's best animal friend has been, and still is for the most part, at the mercy of ignorant quacks. Mr. Hill has done a good service in presenting compactly and clearly what is known about the dog's diseases. The treatise is not an exhaustive one and does not pretend to go very deeply into pathological matters. We might, indeed, criticize it on this account, but it is to be remembered that there is as yet no audience for any profoundly scientific treatise on canine diseases.

The book presents the latest and probably the best account of the subject chosen which is to be found in the English language.

